# Beyond Average: Providers' Assessments of Indices for Measuring Pain Intensity in Patients With Chronic Pain

**DOI:** 10.3389/fpain.2021.692567

**Published:** 2021-08-12

**Authors:** Roberta E. Goldman, Joan E. Broderick, Doerte U. Junghaenel, Alicia Bolton, Marcella May, Stefan Schneider, Arthur A. Stone

**Affiliations:** ^1^Department of Family Medicine, Warren Alpert Medical School of Brown University, Providence, RI, United States; ^2^Dornsife Center for Self-Report Science, University of Southern California, Los Angeles, CA, United States

**Keywords:** pain intensity, pain measurement, mixed-methods research, qualitative research, provider interviews, chronic pain

## Abstract

**Introduction:** Effective clinical care for chronic pain requires accurate, comprehensive, meaningful pain assessment. This study investigated healthcare providers' perspectives on seven pain measurement indices for capturing pain intensity.

**Methods:** Semi-structured telephone interviews were conducted with a purposeful sample from four US regions of 20 healthcare providers who treat patients with chronic pain. The qualitative interview guide included open-ended questions to address perspectives on pain measurement, and included quantitative ratings of the importance of seven indices [*average pain, worst pain, least pain, time in no/low pain, time in high pain, fluctuating pain, unpredictable pain*]. Qualitative interview data were read, coded and analyzed for themes and final interpretation. Standard quantitative methods were used to analyze index importance ratings.

**Results:** Despite concerns regarding 10-point visual analog and numeric rating scales, almost all providers used them. Providers most commonly asked about *average pain*, although they expressed misgivings about patient reporting and the index's informational value. Some supplemented *average* with *worst* and *least pain*, and most believed pain intensity is best understood within the context of patient functioning. *Worst pain* received the highest mean importance rating (7.60), *average pain* the second lowest rating (5.65), and *unpredictable pain* the lowest rating (5.20).

**Discussion:** Assessing *average pain* intensity obviates obtaining clinical insight into daily contextual factors relating to pain and functioning. Pain index use, together with timing, functionality and disability, may be most effective for understanding the meaning to patients of high pain, how pain affects their life, how life affects their pain, and how pain changes and responds to treatment.

## Introduction

Effective clinical care for chronic pain requires accurate, comprehensive, and meaningful pain assessment. It is widely acknowledged that patients' pain experiences are multidimensional, including sensory, affective, and perceptual aspects (1–3). Of the different dimensions of pain assessment, pain intensity is a primary focus in clinical care and pain management to indicate the magnitude of pain, and is meant to describe pain level or intensity (4). Self-reports of pain intensity are typically collected during patient encounters and also represent the primary outcome in most clinical trials of pain disorders (1, 5, 6). Although patient self-report pain ratings using a 0–10 numeric rating scale or a 100-mm visual analog scale are commonly used in clinical practice, improving the degree to which pain assessments provide clinically useful information can facilitate optimal patient care.

Many instruments are available to measure pain intensity. They vary by type of response options, descriptors used to anchor pain ratings (e.g., “pain as bad as one can imagine”), and the reporting period specified (e.g., pain over the past week, past month) (5). A common feature of most pain measures is their focus on *average* level of pain over a period of time. However, a fundamental quality of the pain experience is that pain does not remain at the same level all of the time. Prominent recommendations for core outcome measures in chronic pain clinical trials emphasize the measurement of specific features of pain intensity over time, such as pain maxima, minima, and frequency (1), as secondary outcomes. Additionally, temporal patterns of pain (e.g., episodic, chronic recurrent, constant but fluctuating in intensity) have been described as important to classifying chronic pain (2).

Over the past decades, real-time data collection methods involving Experience Sampling or Ecological Momentary Assessment (EMA) have received increasing attention in pain research. Using EMA, patients rate their momentary pain intensity multiple times per day in their natural environment, which makes it possible to capture temporal features of patients' pain intensity in great detail (7–10). While assessments of specific aspects of pain intensity other than average pain are beginning to be acknowledged in research on chronic pain, to date, it is unclear which temporal indices of patients' pain intensity should be assessed to achieve the greatest utility. Of various pain indices, the *worst* (highest) and *least* (lowest) pain over time have received substantial attention in empirical research, and have been recommended as outcomes in clinical trials (1, 11–14). Additionally, empirical studies suggest that the *amount of time* patients spend in *low* pain or *high* pain represent distinctive features of the pain experience (15–17). Evidence from observational research and clinical trials also highlights the importance of examining pain *fluctuation*, which has been linked to psychosocial outcomes and assay sensitivity (18–23). Finally, studies have shown that the *unpredictability* of shifts in pain [e.g., whether pain occurs after a specific trigger or without warning] is associated with central nervous system performance and functional outcomes (24–26).

These findings suggest that, from an empirical perspective, alternative measures of pain intensity may augment understanding of patients' pain experience and how pain relates to functioning in daily life. However, we know very little about the applied clinical relevance of such assessments, that is, the extent to which they would also augment the information available to clinicians in routine pain practice outside of the research context. This is an important gap in the existing literature because the benefits of utilizing measures that capture alternative aspects of pain intensity levels in patient care depend upon whether they fit the needs and perspectives of those providing medical care to patients.

The present study aims to address this gap. Incorporating stakeholders such as healthcare providers in research to evaluate outcome measures has been strongly promoted by policy makers and regulatory agencies (27–31). In the present mixed-methods paper (which uses data from a larger study that included providers, patients, and regulators), we aimed to investigate providers' perspectives on and ratings of the utility of measures focusing on alternative aspects of pain intensity when evaluating treatment outcomes in chronic pain care. We included a quantitative rating exercise within a qualitative individual interview of healthcare providers. The primary research questions guiding the healthcare provider interviews were: How do providers evaluate the utility of pain intensity assessment in clinical practice? Which aspects of pain intensity are most useful to providers in managing their patients' chronic pain? How do providers value assessments that capture specific aspects of patients' pain levels in addition to (or as alternative to) *average pain* level?

## Materials and Methods

### Eligibility, Recruitment, and Providers

Healthcare providers were recruited for interviews through the American Academy of Pain Medicine (AAPM) mailing list. Providers were selected randomly from the list based on geographic region by targeting zip codes, representing 13 US states and four geographic regions—Northeast, Midwest, South and West. A total of 81 males (56%) and 64 females (44%) across the country were sent participation invitation letters by postal mail. An initial batch of 100 invitation letters was sent; due to low initial response, we used the same method to send a second batch of 45 letters to a new set of providers. All providers were purposively selected based on sex and geographic region. Follow-up phone calls were made to anyone who did not respond to the letter. Eligibility included ability to read and speak English, willingness to provide verbal informed consent, and work role including more than 8 h per week of seeing patients with chronic pain. A threshold of 8 h per week was selected to allow for inclusion of providers who were not exclusively focused on treating patients with chronic pain and treat patients outside of pain specialty settings. The process we used is concordant with purposive sample creation (32) whereby a small sample is selected that includes the diverse characteristics desired in the sample, and recruitment and data collection ceases when data saturation is achieved (33, 34).

### Data Collection

#### Procedures

The study was approved by the University of Southern California Institutional Review Board (UP-15-00228) and informed consent was obtained from all enrolled providers. Participants were sent a reminder email 2 days prior to their scheduled interview with an informed consent information sheet, and a pain index sheet containing seven pain indices and definitions: patients' average pain, worst pain, least pain, the amount of time patients spend in no pain or low pain, the amount of time patients spend in high pain, the extent to which pain fluctuates, and the unpredictability of shifts in pain (Table 1). The indices were selected based on the literature of basic temporal and distributional characteristics of pain that are commonly derived from EMA and other diary methods (7, 10, 35). We note that this list is by no means exhaustive, and more complex temporal features of pain such as the dominance in duration of high vs. low pain states (8) or the autocorrelation of pain intensity states (7) that have been examined as EMA-derived pain outcomes are not considered here. Interviews were conducted by the first author (REG). The semi-structured interviews were audio recorded, lasted between 30 and 45 min, and were professionally transcribed. The initial monetary incentive offered to providers was a $150 gift card, which was later increased to $200 to enhance participant recruitment.

**Table 1:** Pain indices and definitions presented to providers during the interviews.

**Pain index**	**Definition/Explanation**
Average pain intensity over a week	If we take many ratings of a patient's pain intensity during a week, add them up and then divide by the number of ratings, this would give us an average of a patient's pain during that week.
Level of pain intensity when it is at its worst during a week	If we take many ratings of a patient's pain intensity during a week, we could see what a patient's *highest* pain level was. This would indicate the level of pain intensity when it was at its worst.
Level of pain intensity when it is at its least during a week	If we take many ratings of a patient's pain intensity during a week, we could see what a patient's *lowest* pain level was. This would indicate the level of pain intensity when it was at its least.
Amount of time patient spends with no or low pain during a week	This refers to how much of the time during the week a patient didn't feel any or felt very little pain. That is, if we were to take many ratings of a patient's pain intensity, we could figure out the amount of time during a week that a patient had no pain or almost no pain.
Amount of time patient spends in high pain during a week	If we were to take many ratings of a patient's pain intensity during the week, we could figure out the amount of time when a patient had ratings of pain intensity at very high levels.
How much pain intensity fluctuates or changes during a week	If we take many ratings of a patient's pain intensity during a week, we can get a sense of how much a patient's pain intensity varies from moment-to-moment or day-to-day over the week. That is, whether the intensity is more or less constant or how much a patient's pain fluctuates [that is, goes up and down].
Amount of unpredictability of pain levels during a week	This refers to the degree to which a patient's pain intensity changes for reasons that the patient can't identify. If a patient doesn't know when and why his/her pain changes, then a patient's pain levels are unpredictable.

#### Interview

The interview question guide explored how healthcare providers typically collect information about their patients' pain levels; how they view each of the seven pain indices; and which indices might be most useful in their work with chronic pain patients, and why. Core questions were asked of all participants, supplemented by spontaneous probes and follow-up questions. Open-ended questions were followed by structured questions to explore providers' perspectives on and experiences with the seven different pain indices. During this latter part, the interviewer asked participants to talk about each index in terms of the most important/useful pain outcomes of pain treatment, and the most important/useful to them in their work with patients.

Next, the pain measurement concepts sheet was used for rank ordering and rating tasks intended to elucidate the subjective usefulness of each of these indices to providers for characterizing patients' pain (Results for the rank ordering task are presented elsewhere) (10). For the rating task, participants rated each of the indices independently for importance for measuring treatment response, where 0 = no importance and 10 = extremely important. Providers read their ratings aloud for the interviewer to document, and explained in their own words how they made their decisions.

### Data Analysis

Standard quantitative methods were used to analyze the ratings of each of the indices. A repeated-measures ANOVA with one within-subjects factor (importance ratings) was performed to test the omnibus null hypothesis that all pain indices were rated as equally important. Pairwise *post-hoc* comparisons with Benjamini-Hochberg correction (36) to control for inflation of Type 1 error due to multiple (i.e., 21) comparisons were subsequently performed to test for differences in the mean importance ratings between individual indices.

Qualitative interview data were analyzed in iterative fashion, beginning as the transcripts became available and continued through and beyond data collection. In this way, the researchers were able to recognize when they reached data saturation such that no new content or concepts were appearing in the interview data, and data collection should stop. This process resulted in our ceasing data collection after 20 interviews were completed and analyzed.

First, the immersion/crystallization technique (37) for data analysis was used, which involved repeated readings of the transcripts with careful note-taking and team discussions about emerging patterns and themes. We constructed a saturation grid to track patterns as they emerged and to determine when no new information was obtained (33, 34). This process was supplemented with template organizing style analysis (38) where a codebook and coding dictionary were created based on topics and themes identified through individual immersion and discussion among the project team members. This was followed by independent line-by-line coding by two team members using NVivo software (39, 40). Inter-rater reliability was assessed as the coders repeatedly met throughout the coding process to compare and refine their use of codes. Transcripts and code reports were then read again, with discussions among team members to consider alternative interpretations of the data, reconcile conflicting interpretations, and to come to final presentation of results (41–43). COREQ guidelines for reporting qualitative research were consulted during the preparation of this article.

## Results

In this paper, we present findings from analysis of qualitative data from provider interviews, as well as quantitative results of a pain index importance rating exercise providers completed during the interview.

### Participant Characteristics

The 20 provider participants were drawn from four broad regions of the US: Northeast (*n* = 5), South (*n* = 7), Midwest (*n* = 2), and West (*n* = 6). There were 15 MDs, 2 NPs, 1 PA, 1 PhD Psychologist, and 1 PhD Pharmacologist/Toxicologist; 13 males and 7 females, aged 31–65, with mean age of 43.8. Years in practice ranged from 1 to 30, with mean years of 14. The sample size used in this study is consistent with qualitative research design, and our iterative analysis process ensured that saturation was reached (42, 44).

### Providers' Perceptions of the Validity of Standardized Pain Rating Scales

Regardless of which type of index providers in this study favored for use in routine clinical care to measure patients' perceptions of their pain, almost all asked patients to report their level of pain using a 10-point visual analog or numeric rating scale. Nevertheless, providers described multiple problems with this method. Many providers stated that, over time, even if other indicators demonstrated that the patient's pain had improved (e.g., increased function), some patients persisted in reporting their pain intensity at a consistent, high level on the scale. Providers attributed this inertia in pain reporting to a patient's long-established self-identification as a person with a high level of pain.

“And so, they really don't seem to move a lot on the number itself. And part of that is something I of course don't at all understand. But I think that it really has become just more of, ‘I'm an 8.' It's just one of those things.”

Other problems providers cited were patients' lack of literacy regarding use of scales, the idiosyncrasy with which the points on the scale are viewed from patient to patient, and patients' reluctance at times to even designate a point on the scale.

“I think there's a big problem with the scale. A lot of patients just don't understand what it means. Some people, they are in terrible pain, but they will still give you a lower number, and others may not seem to be in such terrible pain, but they always have higher numbers.”

“[Patients] get frustrated when you ask them, ‘What is the lowest pain?'. Sometimes people say, ‘Well, the lowest pain is I don't have pain sometimes at all. And sometimes I have it but it's really bad.' I think it's a very difficult question to answer.”

“They say the pain is higher than it really is, or they say the pain is a 12 or 15. They walk comfortably into the office, and they'll be sitting there breathing normally. So I find a lot of patients, no matter how hard I try to put it in context, don't really understand.”

Providers in this study observed that patients can more easily recall high pain than low pain. Therefore, participants believed that when patients are asked to recall their pain over a period of time up to the present, patients most often focus on the higher pain levels they experienced, regardless of what percentage of the time they endured high pain. In addition, many providers felt that patients tend to “catastrophize” their pain.

“So if they tell me that they have chronic 15- and 12-out-of-10 pain usually, and they're sitting there comfortably in front of me, then I'll kind of dig into it a little deeper and say, ‘Well, what's the worst pain you ever felt?”

Another variable inherent in the use of pain scales for interpreting patients' pain levels is the reason that individual patients are seeing the provider at that time.

“It varies on what they're here for. Are they here to get pain medications? Then they're going to be a 10 all the time. Are they here to get a procedure? Then they may be a little bit lower on the scale. It varies on what I see that they're looking for. So you can ask them where their pain is, but you haven't figured out how to put a meter on that yet.”

Some providers in this study noted that when patients are in pain at the moment of their medical visit, it can be difficult for them to focus on how the pain was different in the preceding period of time: “[Patients are] just trying to make it through the next hour until ‘I get my pills.” Further, many providers explained that patients with chronic pain over time come to relate to their pain as a significant element of their identity, which consciously or unconsciously, they become reluctant to relinquish for a variety of reasons.

“All of this pain and how they relate to it has become part of their story that they tell themselves. If they have been self-identifying as a pain patient in some way for a long period of time, I think simply that having to let that go and move on, in and of itself, is anxiety inducing. And so even if they're doing well they want to hedge their bets a little bit and they want to say, ‘Okay, I'm feeling a little bit better but I am not about ready to say that I'm all the way better or I'm getting better cause what if this goes away, what if this is only temporary? I've been burned in the past.”'

### Providers' Strategies for Assessing Patients' Pain Intensity

Several themes emerged in response to the question about how providers typically collect information about their patients' pain levels. A few providers asserted that they do not start their pain and treatment efficacy assessment by asking about intensity, and instead ask questions such as, “Did your pain get better?” Most providers, however, stated they typically begin by asking patients what their level of pain is at the current moment. Most then ask what their patients' pain has been *on average*, over a past period of time, sometimes unspecified though usually the past 7 or 30 days. Some ask for pain levels on different specific days, or weekends vs. weekdays. Many said they end their inquiry about pain intensity there, although some next proceed to one or more additional pain indices, most often *worst pain* and/or *least pain* over the specified period. As one provider explained:

“When I ask people about low pain first before their worst pain, they don't even answer low pain. They would answer the worst pain. It's just because I think they think if they say the lowest pain first I would not ask about their worst pain. And they would not get the treatment they deserve or whatever. Now what I've started doing is I ask the worst pain first.”

One provider who predominantly provides injections and other pain-relieving procedures explained:

“Unfortunately, we try to boil everybody down into a little pot and it never works with pain because it's so multidimensional. But when we measure pain as one point, the FDA decided it wasn't pain intensity that was important. It was pain relief. So that a patient could say, ‘I feel relief' rather than ‘My pain is this.”'

Many participants stated that pain ratings alone are not sufficient for understanding the patients' experiences of pain. Some ask patients for descriptive words about the pain, and most ask about function and ability/disability in addition to pain ratings. Some providers explained that juxtaposing what a patient was doing at the time of having *worst pain* in the past 7 days with what the patient was doing at the time of having *least pain* is critical for assessing whether the treatment is working. Many provided examples such as if a patient's *least pain* occurs in conjunction with lying on a comfortable couch and *worst pain* occurs when doing a physical task, pain ratings are placed within the context of daily life and can inform treatment decisions. While overall, *fluctuating pain* was rated by providers as among the least useful indices, some said they used this index specifically to ask about context and activity, and then to educate patients about managing their high and low pain levels throughout the day.

Some providers said that when patients with chronic pain succumb to fluctuating pain by avoiding normal daily activities that increase the pain, they do themselves a disservice, and besides treatment “[it takes] a little bit of education. Because we know that it's going to fluctuate. Sometimes catastrophizing, and just fear that the pain's going to get worse if they do anything, and anytime it fluctuates a little bit, [they believe] it's getting worse.” Providers emphasized that however pain intensity is identified, effective treatment is predicated on their own good communication and listening skills.

“Something that I view more about the population [patients with chronic pain] overall is that they don't feel heard and they don't feel believed by people. And whether that's their peers, or they're walking around hurting and they don't have a broken arm, or they're not in a wheelchair. Their life is very difficult, but they *look fine*. And that's a very frustrating experience for them. So to me, [patients' pain reporting] is really about communication, like a way to say, ‘Things are really bad'. It's one of the only ways they think they have to express how bad things are for them because they feel very misunderstood and not heard.”

Some providers said they try to enhance the usefulness of the pain measurement scales by regularly engaging in educating patients about what the scales mean.

“When people think 10, I don't take them at face value, sometimes. I'll say, ‘So you barely got out of bed this morning 'cause you're at a 10?' ‘Oh, well, okay, maybe it's an 8.' So I really educate them on the numbers and the specificity of it. ‘When you say it's the worst pain of your life, explain to me.' So we have a lot of education in my practice and I really reinforce the patients to be involved and make it a team effort.”

Regardless of the pain indices providers preferred, most used these in an effort to gauge how the patient's pain has changed over time and in response to treatment. As one provider stated: “So it's something that we can look and see where they were at. It's not very reproducible *between* people, but for the *same person* it might be indicative of how they're doing today vs. how they've done in the past.” Ultimately, if the patient is receiving treatment for the pain, the goal is to “Look what pain does to disrupt life, and what is that treatment doing for that.”

### Providers' Perspectives on the Pain Indices

**Figure 1:**
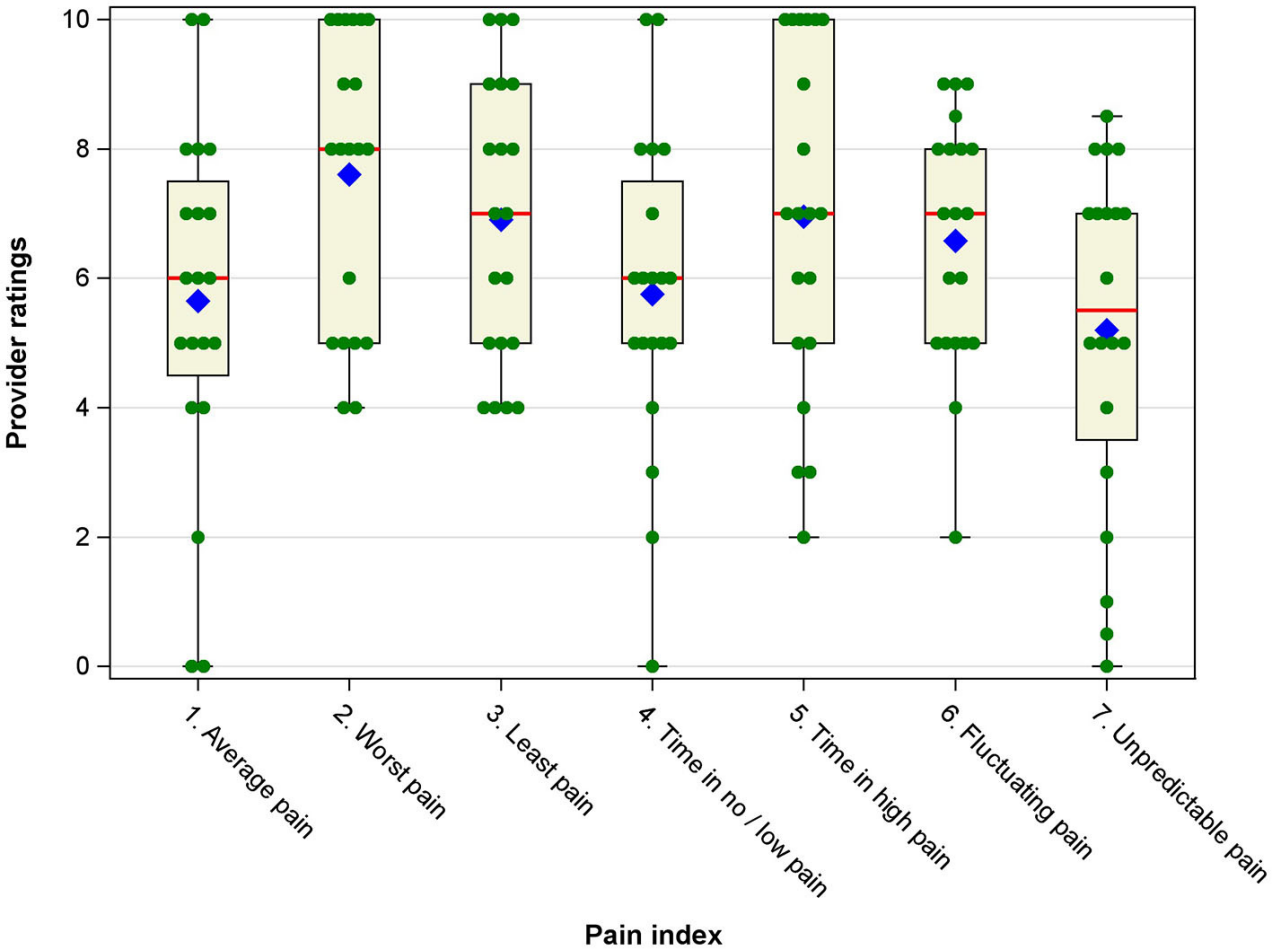
Box-and-whisker plots of provider ratings of importance/usefulness of the pain intensity indices. Blue diamonds represent the mean, red vertical lines represent the median, boxes represent the 25th and 75th percentile, whiskers represent the range of ratings, and green filled circles represent individual provider ratings for each pain index.

Understanding how healthcare providers viewed the importance of the seven pain indices was an essential component of data collection. The individual provider ratings for each pain index are displayed in box-and-whisker plots in Figure 1. Table 2 provides summary statistics. Descriptively, *worst pain* received the highest mean importance rating. *Unpredictable pain* received the lowest rating. *Average pain* received the second lowest importance rating. It is notable that all seven pain indices received mean importance ratings above the midpoint of 5 on the 0–10 scale, suggesting that all indices were deemed somewhat important by providers. In addition, providers varied substantially in the importance ratings of each index, with standard deviations approaching or exceeding 2 scale points, suggesting there was limited consensus among providers about which indices are most and least important.

**Table 2:** Mean [SD] of provider ratings of importance/usefulness of individual pain intensity indices.

**Pain index**	**Mean [*SD*]**
Worst pain	7.60 [2.23]
Time in high pain	6.95 [2.67]
Least pain	6.90 [2.20]
Fluctuating pain	6.58 [1.93]
Time in no pain/low pain	5.75 [2.45]
Average pain	5.65 [2.76]
Unpredictable pain	5.20 [2.66]

*Pain indices are displayed in order of mean importance ratings from most important to least important*.

In statistical analyses, a one-way repeated-measures ANOVA yielded a significant omnibus F-test, [*F*(6, 14) = 3.11, *p* = 0.007], indicating significant differences in the mean importance ratings. *Post-hoc* pairwise comparisons showed that *worst pain* was rated significantly more important than *time in no/low pain* (*d* = 0.64, *p* = 0.040) and *unpredictable pain* (*d* = 0.94, *p* = 0.01). In addition, *least pain* and *fluctuating pain* were rated significantly more important than *unpredictable pain* (*d* = 0.64, *p* = 0.040, and *d* = 0.59, *p* = 0.048, respectively).

In the qualitative interview component, providers claimed to use *average pain* exclusively or at least most often because they knew it to be the most commonly-used index in clinical care and clinical trials. Some admitted they had never considered other ways of measuring pain intensity until the six additional indices were outlined during the study interview. Despite their consistent use of *average pain*, providers described numerous problems with it which were reflected in the ratings. They stated that patients misconstrue the meaning of average to include the level of pain experienced most frequently (i.e., mode), rather than the arithmetic mean. Others asserted that since high pain is more memorable than low pain, the reported average will be pushed artificially higher. One provider explained how patients become irritated by the request to report average since patients see their pain as unique, not “average.” Other providers said patients insisted that their pain was far worse than “average.” These misconstrued ways of responding about *average pain* would corrupt the meaning and interpretation of this index if providers assumed patients were referencing the *average pain* level over the prescribed period.

Some providers explained that recall of specific pain levels during the designated time period was a problem even if patients knew how to calculate average. One provider tried to mitigate this problem by having patients keep pain logs: “And then we would go ahead and take their score, average it by the number of readings, and then we say, ‘See, your average pain is 5.' [And the patient would respond], ‘Oh no, it's got to be an 8'. So we stopped doing that.” Some providers who acknowledged the inadequacy of *average pain* ratings still felt that seeing how patients' reported averages went up or down over time is useful, no matter how the patients conceptualize the concept of average, and so the index is still in common use. As a provider claimed, “I've found most value in the average because I think it's taking out jagged edges. I find the average is something that is going to give you a better curve with less disturbance in it.” Some claimed that *average pain* may still be the best indicator of pain intensity over time, but not in the way it is currently used: “I think it would be education on the patients' part, my part, making sure we're all on the same page.”

Many providers said they valued the *least pain*, and to a lesser extent *time in low pain*, indices for the information these provide about how medication or behavioral treatment is working, and for the success and relief good values imply for the patient. A few providers stated this preference in terms of “putting a positive spin” on the patient's experience of chronic pain. In contrast, some noted that if a patient already has considerable time in low pain there is not much for the provider to do to help the patient, so the index is less useful. Many providers in the study stated that these two indices are difficult to use with patients. They attributed this evaluation to factors they have observed in their practice: patients' inherent bias toward remembering more clearly their worst pain and time in high pain; patients' tendencies to “catastrophize” their pain; the centrality in patients' minds of lowered functional capacity due to those times in high pain; and patients' reluctance to admit or talk about any lessening of their pain for fear that providers would become distracted from or not take seriously their reports of accompanying periods of high pain. Providers said they believed that the patients' primary goal is to keep their providers' “attention on the pain.” In addition, at the time of the interviews there was increasing media attention across the US about the burgeoning prescription opioid crisis, and providers speculated that the resulting environment of heightened pressure to decrease opioid prescribing may impact patients' urgency to justify continuation of medication: “In some cases they feel that if they don't continue to rate their pain high, maybe you're going to say, ‘You don't need all this medication.”'

“They are pretty wise about the number they *need to give*, for it to be noteworthy enough to a provider. So, that individual may be more likely to report a 7, an 8, a 9. I have many people that will say, ‘My 8's like anybody else's 20.' Everyone thinks that theirs is the worst.”

“I realize that there's that fear that if they say they're doing better, ‘Oh good, then it's time to reduce their pain meds.'. So they absolutely do come in with the worst pain, 9, and they're not looking like they're about to die. I suspect that's what they're concerned about, that we're going to take their pain medication away and they're going to be miserable and not able to work or function or have a good quality of life.”

Some providers offered that this reaction could backfire as patients who persist in reporting inflated pain levels may lead providers to reduce or completely de-prescribe seemingly ineffective medication.

“But when you sit down and say, ‘Look, this is not working for you. You've been seeing me for months and every time you come in here your pain's a 9 or a 10. That tells me that what we're doing is not working and now we need to reassess whether this is actually helping you. And being that it's so high all the time, I can't keep you on this medication.”'

Given providers' views that patients are averse to reporting *least pain* and *time in no or low pain*, some explained that they avoided these indices in clinical practice even though they themselves felt they were good indicators of pain intensity. Providers who supplemented *average pain* with other indices said they do use *time in no or low pain* to ascertain how a patient's ability to function (“what they can do”) has changed since the previous medical visit. Some said they felt that a patient's reporting of low pain is extremely significant since it is so much less memorable than high pain. However, given the difficulties of having patients focus on low pain, more providers used *worst pain and time in high pain* to understand what a patient can and cannot do, and they gave these two indices high importance ratings.

“If we are able to reduce the amount of time in high pain, I think that would be a useful measure, and even if we're not able to reduce the average pain but are able to reduce the amount of time in high pain I think that would give patients a better quality of life. And I think that would be a useful thing to follow, and especially if we were to show medical necessity for our treatment, that we're reducing the time in high pain and it's improving quality of life.”

Providers asserted that *unpredictable pain* is especially debilitating for patients, impedes patients in planning activities, and has a high emotional toll. However, providers explained they rated the index as least useful since they are unable to adequately treat these unexplainable onsets of pain.

### Inextricability of Pain Intensity and Function

The importance of different pain indices for understanding patient functioning arose spontaneously throughout the interviews, with most providers stating that, ultimately, it is functionality that patients value and seek. Providers emphasized the importance of understanding the direct effect of pain intensity on the patient's functioning, and interpreting the meaning of each measurement in relation to what the person was doing at the time the measurement refers to. “It's more important to identify when the pain is at its worst and *what's going on at that point*, and when the pain is at its least *and what's going on at that point*. Sure, you can average those numbers, but I'm not quite sure if patients would think about it like that.” Providers emphasized that highlighting function is particularly critical for patients with chronic pain, because these patients will likely live with some level of pain into the future.

“When I talk to my patients about outcomes, I tell them that we are trying to improve function. We may not make it go away completely because most of the pains are chronic, they don't go away, but we are trying to improve the quality of life and improve the function. That's the goal.”

“I have a little graphic that I show people. You're trying to make life feel bigger so pain feels smaller by comparison. Your pain may not change at all, and that's just the truth.”

Providers, therefore, emphasized that the most reasonable treatment goal is to increase functioning, which necessitates educating patients so as to minimize their tendency to give in to the pain and decrease their activity.

“If the person has a memory of their pain coming down, that suggests that they're learning from what I'm trying to teach them. What I'm also trying to frame for them is that pain goes up and down. Increases and decreases in pain really have not a lot of meaning with respect to anything in a chronic pain patient being wrong. So, [I tell patients] ‘You should continue with your activity program. Continue with your therapy. Yes, your pain is gonna be from time to time worse, but that doesn't mean you're causing harm.”'

## Discussion

Accurate assessment of pain intensity is a basic necessity for gauging change in pain levels, providing adequate treatment, and communicating with patients about their pain (2, 45). Researchers have become increasingly interested in understanding pain intensity as a dynamic phenomenon (7, 8, 20). In fact, the ability to quantify, predict, and possibly influence dynamic aspects inherent in the ebb and flow of pain in patients' daily lives has been described as a paradigm shift in pain research (46). However, less is known about the extent to which assessments capturing specific temporal aspects of pain would augment the information available to clinicians in routine pain practice. This interview study with providers who care for patients with chronic pain found that, not surprisingly, *average pain* continues to be medical providers' most commonly-used index. Despite common usage, providers did not provide quantitatively high ratings of the importance or usefulness of the *average pain* index. Their reasons included patient confusion about the meaning of average and patients' inability to accurately recall pain levels.

Many participants complement their use of the *average pain* index with questions assessing *worst pain* and/or *least pain*, or *time in high pain* or *low/no pain*. There was little consensus among providers about which index is most useful or important, although overall, *worst pain* was rated highest among the seven indices, and *unpredictable pain* was rated lowest. As others have found (1, 2), *worst* and *least pain* are considered useful to better understand temporal fluctuations or to calculate an average. Interestingly, while *least pain* and *time in low/no pain* were believed to be important, providers found it challenging to focus patients' attention on these and so they may not be feasible indices to use in routine pain assessment.

Our finding that *worst pain* was rated as most important is interesting in view of the US Food and Drug Administration's (FDA) recommendation to use *worst pain* ratings as the primary outcome in drug clinical trials (14). It is possible that providers were aware of the FDA recommendation when making their importance ratings. Regardless, the perceived importance of *worst pain* was supported by this study, especially when coupled with information about patients' activities to better understand potential contributors to pain exacerbations.

The *fluctuating pain* index was rated as only moderately important. However, increasing empirical evidence supports the idea that identifying pain level variations may be an important clinical target. For example, momentary pain fluctuations have been found to relate to affective distress and activity limitations (47), and individuals with greater pain variability have shown higher depression levels and lower self-efficacy for pain management (20). Pain variability may hold promise for informing clinicians about potential barriers to successful adjustment and management (18, 21, 23, 48). Our participants recognized that unpredictable pain can be extremely distressing for patients. However, they were reluctant to ask about it because of their overall goal to control the pain, which is difficult for unpredictable shifts in pain when the reasons are not known.

Providers in our study also emphasized that the importance of different aspects of pain intensity must be understood in the context of its impact on patient functioning. This is in line with recommendations for “core outcome measures” for chronic pain by the Initiative on Methods, Measurement, and Pain Assessment in Clinical Trials (IMMPACT) (1). A survey of patient stakeholders showed that patients considered a variety of functioning domains [e.g., emotional well-being, enjoyment of life, fatigue] as highly important for evaluating the consequences of their chronic pain (49). Different aspects of pain intensity may have interactive or cumulative effects on specific facets of patient functioning. For example, in a recent study, we found that pain variability, worst pain levels, and the time chronic pain patients spent at high levels of pain uniquely related to patient physical and social functioning above the effects of average pain (9). The present study supports the importance of recognizing the pain-functioning linkage from a clinical pain management perspective.

Finally, patients' ability and willingness to properly use the pain rating scale was a consistent provider concern in our study. Prior qualitative (50) and quantitative (51) research with chronic pain patients showed that the ostensibly simple task of completing standardized pain ratings is often approached idiosyncratically. The task to provide recall pain ratings over extended periods of time further adds to the complexity of obtaining accurate pain summary ratings (52). Pain rating trainings (53), as well as clearer instructions and more precise descriptions of scale anchors and recall periods (52), might improve pain rating accuracy. Whether these could be implemented in routine clinical care should be explored.

This study has several limitations. Our sample consisted predominantly of MDs, and the results may be different across different professional backgrounds or areas of specialty. Even though the invitation letters were sent through the AAPM mailing list, invitations to participate in the study were unsolicited, and only 14% of providers responded, which may have biased the results due to self-selection effects. Nevertheless, our sample was geographically diverse and robust in that we were able to stop recruiting interviewees after having interviewed 20 providers because our iterative data analysis process allowed us to identify that we had reached data saturation. Furthermore, clinicians generally had been treating patients for a considerable amount of time (average = 14 years). Providers who are newer to the field may not hold the same views. Additionally, even though our sample size was consistent with prior qualitative work, it should be considered small for the quantitative analyses. Larger samples could examine the hierarchy of preferences for different pain measures and enable subgroup analyses to compare preferences based on clinicians' professional background, years in practice or area of specialty. Along similar lines, our sample consisted predominantly of health care providers with prescriptive authority; an interesting direction for future research would be to compare the preferences between providers with and without prescriptive authority. Finally, in future research, it would be valuable to compare the perspectives of healthcare providers with those of patients with chronic pain. Understanding how patients' perspectives might relate to providers' views could be particularly valuable when it comes to assessments of pain intensity because prior research has shown that patient and provider ratings of patient pain intensity do not necessarily correspond with one another (54–56). We note that we had originally attempted to compare the views of providers and patients as part of this study. Unfortunately, the patient interviews did not provide sufficiently detailed and nuanced information to pursue meaningful qualitative analysis in this group. It is well-possible that interview scripts that are specifically tailored to patients and their personal experiences with pain in daily life (rather than probing patients for their opinions about specific pain measures, as was attempted here to maximize comparability between interview scripts for patients and providers) would have yielded richer qualitative patient data.

## Conclusions

The main goal of the present study was to examine whether specific aspects of patients' pain intensity other than average pain would be viewed as useful by providers. Most providers in our study agreed that inquiring about multiple aspects of pain intensity could augment patient evaluation in clinically relevant ways. They described how additional indices beyond or instead of *average pain* (particularly *worst pain* and *least pain*) would constitute a more effective strategy for pain measurement. Providers also mentioned the benefit of including contextual information about timing, function, and disability for enhancing understanding of patients' responses to treatment and for understanding the meaning to patients of high pain, how pain affects their life, how life affects their pain, and how pain changes and responds to treatment. Provider preferences are just one important aspect in a comprehensive effort to identify the relevance of alternative pain intensity measures. Future studies should therefore test the usefulness of soliciting different types of pain intensity information directly in clinic settings to evaluate the practical gains for routine care. Additionally, more research is needed to evaluate whether different aspects of pain intensity are differentially impacted by treatment, and whether assessment of multiple aspects of pain intensity could contribute to treatments that are more closely tailored to the needs of individual patients.

## Data Availability Statement

The raw data supporting the conclusions of this article will be made available by the authors, without undue reservation.

## Ethics Statement

The studies involving human participants were reviewed and approved by University of Southern California Institutional Review Board (UP-15-00228). The ethics committee waived the requirement of written informed consent for participation.

## Author Contributions

RG conducted interviews, data analysis, and took the lead on drafting the manuscript. JB contributed to study conceptualization and to drafting the manuscript. DJ contributed to study planning and to drafting the manuscript. AB conducted interviews, data coding and qualitative data analysis, and contributed to drafting the manuscript. MM conducted interviews, data coding and qualitative data analysis, and contributed to drafting the manuscript. SS contributed to study conceptualization, conducted data analysis, and contributed to drafting the manuscript. AS conceived of the presented idea, supervised the conduct of the study, and contributed to drafting the manuscript. All authors contributed to the article and approved the submitted version.

## Conflict of Interest

AS is a Senior Scientist with the Gallup Organization and a consultant with IQVIA and Adelphi Values, Inc. The remaining authors declare that the research was conducted in the absence of any commercial or financial relationships that could be construed as a potential conflict of interest.

## Publisher's Note

All claims expressed in this article are solely those of the authors and do not necessarily represent those of their affiliated organizations, or those of the publisher, the editors and the reviewers. Any product that may be evaluated in this article, or claim that may be made by its manufacturer, is not guaranteed or endorsed by the publisher.
